# Intraocular ossification. Case report

**DOI:** 10.22336/rjo.2017.12

**Published:** 2017

**Authors:** Ciprian Maftei, Horia Tudor Stanca

**Affiliations:** * “Prof. Dr. Agrippa Ionescu” Clinical Emergency Hospital, Bucharest, Romania; ** “Carol Davila” University of Medicine and Pharmacy, Bucharest, Romania

**Keywords:** intraocular ossification, osseous metaplasia, bone formation

## Abstract

**Objective:** To report a case of intraocular ossification, describe its particularities and review some of the pathogenesis theories.

**Methods:** We described the case of a 31-year-old woman with a history of perforating trauma ten years before, who presented in our clinic for right eye pain. The patient wanted a cosmetic improvement so an evisceration was proposed. An intraocular hard yellowish mass, which had a histopathological examination, was found intraoperatively.

**Results:** We diagnosed the case as an intraocular ossification, based on the medical history and histopathological specimen examination, which proved to be an ossified structure.

**Conclusions:** In spite of a rare occurrence, our case emphasized the theory that trauma and subsequent neurogenic inflammation could lead to osseous metaplasia.

## Introduction

Eyeball structures ossification is a rare type of metaplasia. Chronic inflammation, trauma, or a long-standing retinal detachment can be an etiologic factor for the heterotopic bone formation. We reported a case of a 31-year-old woman with a history of perforating trauma ten years before, who complained of right eye pain and wanted a cosmetic appearance improvement. During the evisceration, a hard, yellowish mass was discovered in the eyeball. We diagnosed the case as an intraocular ossification, based on the histopathological specimen examination, which proved to be an ossified structure. 

Ectopic bone formation can be found in any soft, highly vascularized tissue, but has a rare intraocular occurrence. In a study conducted by Finkelstein and Boniuk [**1**] on 2486 enucleated eyes, an intraocular ossification was described in only 119 (4.8%) cases. An association between ectopic ossification and long-standing retinal detachment, chronic inflammation, phthisis bulbi, microphthalmia, buphthalmos, or some intraocular tumors [**2**-**4**] was found. A case of intraocular ossification was reported and some of the theories about it were reviewed.

## Case report

**Fig. 1:**
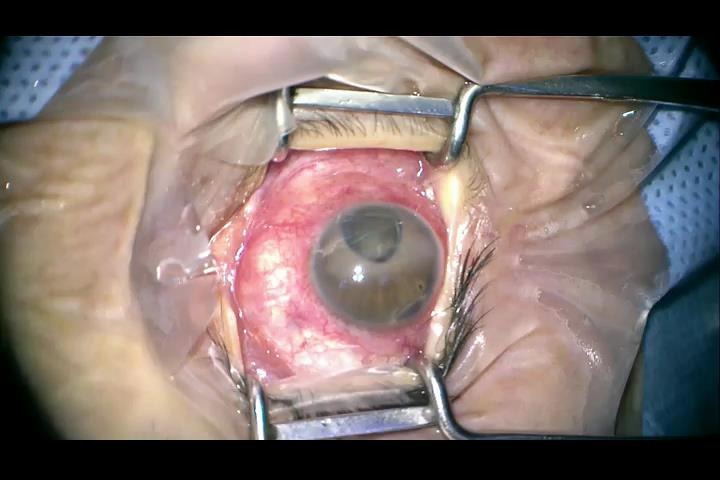
Intraoperative aspect of the anterior segment

A 31-year-old woman presented to our clinic complaining of right eye pain and cosmetic appearance. She had a history of perforating trauma of the right eye due to a car crash ten years before. At that time, she underwent vitrectomy for rhegmatogenous retinal detachment and had several reattachment procedures over the years. On the eye exam, the right eye had no light perception, the right cornea had a central epithelial defect, with fine dust-like subepithelial deposits, emulsified silicone oil in the anterior chamber, a round, nonreactive pupil and corectopia, and it revealed postoperative aphakia (**Fig. 1**). The fundus examination of the right eye revealed full-thickness fixed retinal folds in three quadrants and subretinal strands. The left eye was normal. 

**Fig. 2 a,b:**
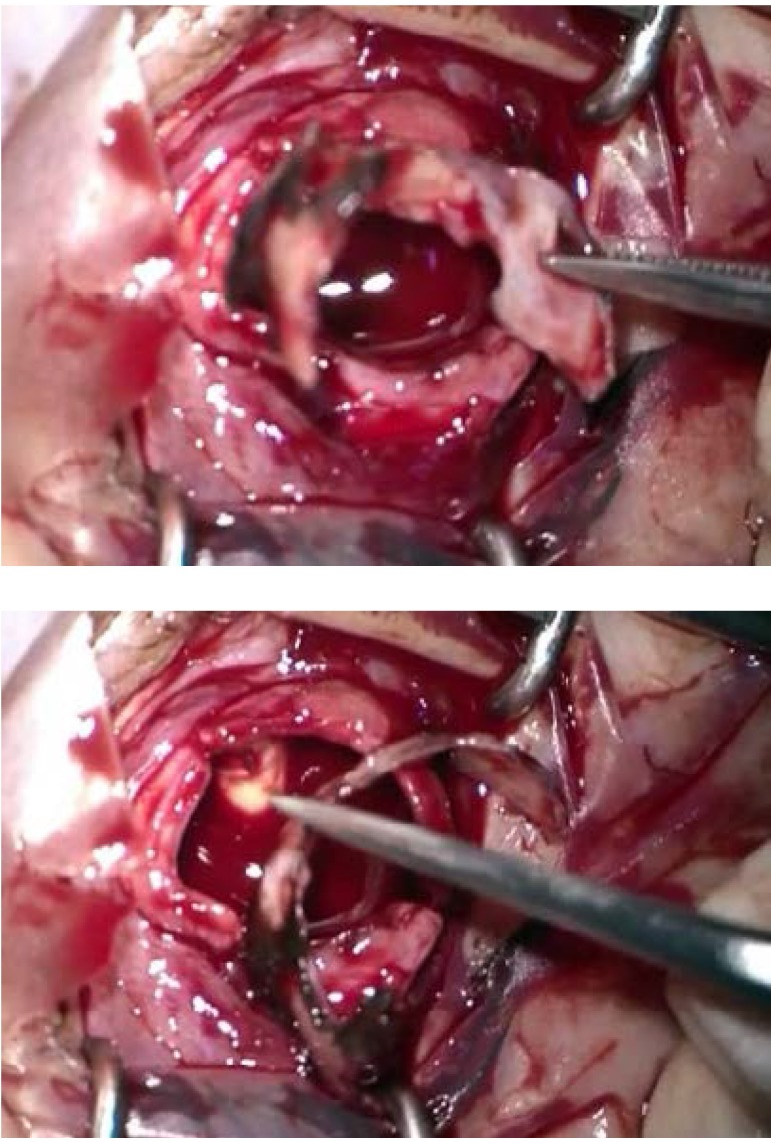
Intraoperative aspect of the intraocular mass

A standard evisceration technique with a polymethylmethacrylate implant was used. When the eyeball content was removed, a hard mass could be palpated covering the temporal inner sclera. With a successive dissection, a hard, white, 30 x 3 x 1 mm mass was exposed (**Fig. 2 a,b**). It was difficult to distinguish the origin of the mass considering the medical history. We had a histopathological specimen evaluation done, which described an ossified structure with areas of thin retinal tissue attached that could be a posttraumatic ossification.

## Discussion

Intraocular abnormal bone formation is an entity that occurs in degenerative tissues, chronic eye disease, tumors, or congenital disorders [**1**-**6**]. Generally, there are two types of osteogenic precursor cells that induce ossification: one can be determined, found in the bone marrow stroma; others are inducible, found in the circulating blood and the connective tissue framework of many other tissues [**7**]. Inducible osteogenic cells need an agent to induce bone formation, which could be cells from the retinal pigment epithelium (RPE) or some types of morphogenic multifunctional cytokines [**8**,**9**]. The retinal epithelium cells in the eye are considered to be pluripotent and have the capacity to differentiate the mesenchymal phenotype, including fibroblasts and osteoblasts which are osteogenesis inducing cells [**1**,**10**].

Intraocular ossification has an incidence reported in large series of enucleated eyes, that varies from 5% to 18%, but there are unpublished studies that report an occurrence rate of more than 38% [**1**,**11**]. The osseous metaplasia is diagnosed differently depending on the date of onset and the examination methods [**12**]. Generally, the studies found that a 10 to 20 year period is needed. In some rare cases, an intraocular bone is formed in less than two years after the initial injury [**1**,**11**]. Choroidal ossification can be histopathologically diagnosed one year after the ocular trauma, but it needs 10 to 20 years to be radiologically identified [**13**]. The correlation between age and the etiological factors of this condition was pointed out in one of the studies. Trauma was the leading cause for the 10 to 50 years old age group, while for the 51-90 years old group, the inflammation was the leading etiological factor [**5**].

The first studies reported the site of intraocular ossification only external to the neurosensory retina [**1**,**5**]. With the advance of surgical techniques, it became possible to conduct histopathological studies on epiretinal membranes. Such studies reported osseous metaplasia in epiretinal membranes obtained at vitreoretinal surgery [**14**,**15**]. Now, we can assume that the location of heterotopic bone can be pre-retinal, sub retinal or in both location and in some certain conditions there could be intraretinal ossification [**11**]. 

Complex pathogenic mechanisms are suggested for every different site of intraocular ossification. The principal source of fibrous and osseous metaplasia appears to be retinal pigment epithelium [**9**]. Growth differentiation factor-5 (GDF-5), bone morphogenic protein-7 (BMP-7), and transforming growth factor beta-1 (TGF β1) are multifunctional cytokines that have important roles in bone formation [**9**]. A model for bone formation was proposed in a study conducted by Toyran S et al. [**9**]. Chronic end-stage eye disease is often accompanied by intraocular inflammation. The inflammatory cells release interleukin-1 (IL-1) or tumor necrosis factor alpha (TNF-α), stimulating the RPE to produce TGF β1 and BMP-7. TGF β1 triggers epithelial-mesenchymal transformation of RPE cells into RPE fibrous metaplasia. BMP-7 inhibits this transformation by counteracting the effect of TGF β1. Additionally, BMP-7 promotes the transformation of metaplastic RPE into osteoblasts. It is likely that GDF-5, which was co-localized with BMP-7 in areas of RPE metaplasia, also stimulates osseous metaplasia.

Preretinal ossification occurs after the migration of retinal pigment epithelium from the sub-retinal space to the retinal surface, along the back surface of the detached retina (through retinal breaks) [**14**,**15**]. Bone formation could occur within the fibrous membranes or the proliferating vitreoretinal mass, suggesting the possibility of multi-directional metaplasia of the retinal pigment epithelial cells [**11**].

Drusen are an abnormal accumulation of extracellular material in Bruch’s membrane that is suggested to be an important step in the transdifferentiation of retinal pigment epithelium following retinal detachment [**16**]. As drusen have various morphologies, there is a possibility that some components within to act like an inducing agent for osteogenesis in earlier stages. 

A different possible theory suggested by Munteanu M et al. [**12**] describes the ossification of the choroid. Some of the factors that cause bone formation within the choroid are BMPs, growth factors, and in particular, pericytes and/ or circulant mesenchymal stem cells (MSCs). The pluripotent MSCs can differentiate between different cell types like osteoprogenitor cells that secrete bone matrix. Bone matrix regeneration and remodeling leads to the formation of spicules and thereafter osseous trabeculae which, by interconnection, generate primary spongy bone, later replaced by lamellar bone. The histopathological aspect of ossified choroidal tissue reveals a spongy type, consisting of osseous lamellae, osteocytes, bone canaliculi, and adipose tissue. This lamellar bone structure supports the hypothesis of endoconjunctive/ desmal ossification, without passing through the cartilage phase. This theory seems to explain more suitable our findings – a hard mass with a semicircular configuration, perfectly adapted to the eye wall and located between the retina and the sclera.

To summarize, intraocular ossification is a rare finding, with complex pathogenic mechanisms not entirely understood. Specialists came to an agreement that chronic inflammation, posttraumatic neurogenic inflammation, bone morphogenic proteins, drusen components, and the differentiation of mesenchymal stem cells are parts of the pathogenesis.

**Financial Disclosures**

None of the authors has any financial or proprietary interests to disclose.
